# Evaluation of Transition Metal Complexes of Benzimidazole-Derived Scaffold as Promising Anticancer Chemotherapeutics

**DOI:** 10.3390/molecules23051232

**Published:** 2018-05-21

**Authors:** Afzal Hussain, Mohamed F. AlAjmi, Md. Tabish Rehman, Azmat Ali Khan, Perwez Alam Shaikh, Rais Ahmad Khan

**Affiliations:** 1 Department of Pharmacognosy, College of Pharmacy, King Saud University, P.O. Box 2457, 11451 Riyadh, Saudi Arabia; afihussain@ksu.edu.sa (A.H.); malajmii@ksu.edu.sa (M.F.A.); m.tabish.rehman@gmail.com (M.T.R.); alamperwez007@gmail.com (P.A.S.); 2 Department of Pharmaceutical Chemistry, College of Pharmacy, King Saud University, P.O. Box 2457, 11451 Riyadh, Saudi Arabia; azmatbiotech@gmail.com; 3 Department of Chemistry, College of Science, King Saud University, P.O. Box 2455, 11451 Riyadh, Saudi Arabia

**Keywords:** metal complexes, DNA binding/cleavage, cytotoxicity, cell migration/adhesion, apoptosis, toxicity profile

## Abstract

Three new transition metal complexes, Cu(II) **1**, Co(II) **2**, and Zn(II) **3** with ligand “bimnap” derived from 1-methyl-2-aminobenzimidazole and 2-hydroxynapthaldehyde were synthesized and characterized. The structure of the ligand was determined by single X-ray crystallography. All the three complexes, **1**–**3,** were examined for the mode of interaction with biomolecule viz., calf thymus-DNA (CT-DNA) using various spectroscopic methods. The nuclease activity was performed against pBR322 DNA that exhibited concentration-dependent degradation of the nucleic acid. The mechanism of DNA cleavage was studied by the electrophoretic pattern in the presence of the radical scavengers. Also, the complexes **1**–**3** were analyzed for groove binding affinity. Moreover, in vitro cytotoxicities of the complexes **1**–**3** were tested against the five human cancer cell lines, i.e., HeLa, SK-MEL-1, HepG2, HT108, and MDA-MB 231. Also, the cell adhesion and migration properties upon treatment of cell lines with complexes **1**–**3,** and consequently, their cell death pathway via apoptosis and necrosis were analyzed. Further, complexes **1**–**3** were studied in vivo for their toxicities and tolerabilities in mice. In sum, the complexes **1**–**3** showed merits of an effective anticancer agent in cell lines–based study while minor side effects were observed in vivo.

## 1. Introduction

Even after the significant development of anticancer drugs, cancer is still a major cause of death worldwide. The primary concern in the treatment of cancer is drug resistance and the adverse side effects of the drug. Metallodrugs’ potential as anticancer agents continues to be one of the fascinating areas of pharmaceutical research since the serendipitous discovery of cisplatin and its analogs. However, the drug-associated adverse side effects and resistance hinders the use of these drugs [1,2,3]. This opens up new vistas for the researchers to go for new metal-based chemotherapeutical compounds/complexes. Numerous metal cores have been explored as a replacement of the platinum-based therapeutics. In this endeavor, transition metals are the most investigated class of elements predominantly because of their biocompatibility in the living system and their endogenous presence in living system as co-factors in several enzymes. Another significant property of transition metal-based therapeutics is their ability to mimic natural compounds with low toxicities. Moreover, the propensity of metal-based compounds to efficiently bind DNA and hence leading to its cleavage is one of the hallmarks of an effective anticancer drugs like cisplatin [4,5,6]. The transition metals such as Cu, Co, Zn, Ru, Fe, Ag, and Au are considered to be promising candidates for designing of such therapeutic agents [7].

Among these metals, copper draws the paramount importance due to its oxidative and bio-essential properties [8,9,10,11,12,13]. Copper is present in various metalloproteins such as hemocyanin, plastocyanin, and ceruloplasmin [14,15], and also acts as cofactor in enzymes such as cytochrome C oxidase, DNAzymes, nitrous-oxide reductase, nitrite reductase, laccase, etc. [16]. Copper also plays an essential role in the neurotransmission and cellular respiration processes. Similarly, zinc is also an important (in fact the most abundant) trace element found in human body. It plays a pivotal role in the catalysis, structure of proteins, cell metabolism regulations, metallo-enzymes, and rewinding of the DNA [17,18,19,20]. Many researchers have observed a direct relationship between cancer cell progressions with zinc deficiency [21,22]. Zinc also plays a prime role in gene regulation, DNA synthesis, and induction of apoptosis [23,24]. The other transition metal of paramount significance is cobalt; an essential element, less toxic and integral constituent of the vitamin B12 (cyanocobalamin), formation of RBCs, nerve tissue maintenance and also acts as a cofactor for various enzymatic systems. It is well known that anticancer chemotherapeutics largely leads to DNA damage, inhibits proliferation of the cells, and causes apoptosis [25,26].

The interaction of potential drug candidates with biomolecules like DNA is quite fascinating. Most of the metal-based anticancer drugs are targeted to bind the grooves of DNA through coordinate and non-covalent mode of interactions. Such interactions are mediated through π-π stackings, electrostatic interactions, and hydrogen bonds. The nature of interactions between DNA and metal-based anticancer drugs depends on the organic moieties attached to the metal center [27]. Therefore, the organic framework around the metal core plays a very imperative role and dictates the mode of binding. Thus, designing of the organic moieties is of key significance for clinical application. In lieu of this background, benzimidazole constituent is most important, as it is present as a constituent in the Vitamin B12. This moiety possesses potential to coordinate with the metal, gives planarity to the drug candidate, and mimics imidazole functions in proteins.

Herein, we have designed, synthesized, and characterized new benzimidazole-derived Schiff base and its ternary metal complexes, namely copper(II) complexes (**1**), cobalt(II) complex (**2**), and zinc(II) complex (**3**). The binding of these complexes **1**–**3** with DNA was studied using UV absorption and fluorescence emission techniques. Moreover, the DNA nuclease activity and the mechanistic pathways of complexes **1**–**3** were analyzed using concentration dependent pBR322 DNA cleavage. Further, the cytotoxicity of complexes **1**–**3** was studied against 5 different human cancer cell lines namely, HepG2 (Liver), SK-MEL-1 (Skin), HT018 (Colon), HeLa (Cervical) and MDA-MB231 (Breast). Furthermore, cell adhesion and cell migration phenomenon of the cancer cells upon treatment with the complexes **1**–**3** were also examined. Moreover, the apoptotic potential of complexes **1**–**3** was evaluated using flow cytometry. Lastly, the in vivo toxicity profile of complexes **1**–**3** was determined on the major organs of mice.

## 2. Results and Discussion

### 2.1. Synthesis and Characterization

**Figure 1:**
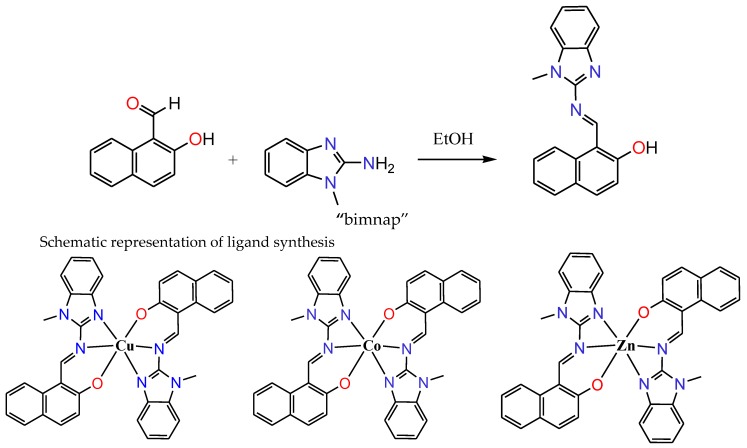
Schematic representation of the ligand synthesis and proposed structures of the Cu(II) (**1**), Co(II) (**2**), and Zn(II) (**3**) complexes.

The new tridentate organic ligand “bimnap” has been synthesized by reacting 1-methyl-2-aminobenzimidazole and 2-hydroxynaphthaldehyde at 80 °C for 2 h in ethanol, leading to the formation of Schiff base (Figure 1). The reaction mixture was kept for slow evaporation, and after 2 days, orange coloured single crystals were obtained. X-ray crystallographic study revealed the formation of the tri-dentate structure of the ligand, which was further characterized using various spectroscopic studies and elemental analysis. The results obtained by all these techniques are consistent with the structure (for detail see experimental section). By taking the ligand “bimnap” in 2:1 ratio with metal salts namely, Cu(CH_3_COO)_2_, Co(CH_3_COO)_2_, and Zn(CH_3_COO)_2_, complexes **1**–**3** were prepared, respectively (Figure 1). All three complexes **1**–**3** were isolated, washed and dried. All the three complexes were isolated in reasonably good yields. Several attempts were made to grow a suitable single crystal of complexes **1**–**3** for the X-ray crystallography but could not be achieved. All the complexes **1**–**3** were characterized using spectroscopic techniques and elemental analysis that was found in good agreement with the proposed structures. Molar conductance values for all the three complexes **1**–**3** which was 1 × 10^−3^ M in DMSO were found to be in the typical non-electrolytic range, thus confirming the non-ionic nature of the complexes.

FT-IR spectra of the ligand showed characteristic signals of aldimine stretching vibration ν(C=N) at around 1622 cm^−1^ (Figure S1). Upon complexation with metal centers, it gets shifted and appeared in the range of 1618–1615 cm^−1^ thus confirming the coordination of metal centers with the C=N (Figures S2 and S3). Also, the stretching vibration of ν(C=N) associated with benzimidazole ring which appeared at 1567 cm^−1^ in the free ligand, showed a significant shift in the region of 1577–1571 cm^−1^ thereby confirms the coordination of benzimidazole ring with metal centers (Figures S1–S3). Furthermore, a new band associated with M-O and M-N appeared at 567–555 cm^−1^ and 463–448 cm^−1^, respectively. Thus, the FT-IR results stand parallel with proposed structures.

The ^1^H-NMR of the ligand exhibited the signal associated with free ligand ‘bimnap,’ and the characteristic signal appeared at 10.36 ppm as a singlet. The aromatic signals lie in the region of 7.51–7.16 ppm and the N-CH_3_ at 3.84 ppm and small broad signal for Ar-OH at 3.56 ppm (Figure S4). On complexation with zinc metal (**3**), the characteristic signals of aldimine proton get de-shielded and lie at 9.69 ppm, similarly shift in the aromatic protons were also observed and appeared in the 8.69–6.56 ppm range and the N-CH_3_ protons as a singlet at 1.90 ppm (Figure S5). The ^1^H-NMR spectrum lies in the agreement with the proposed structure.

### 2.2. X-ray Crystallography

**Figure 2:**
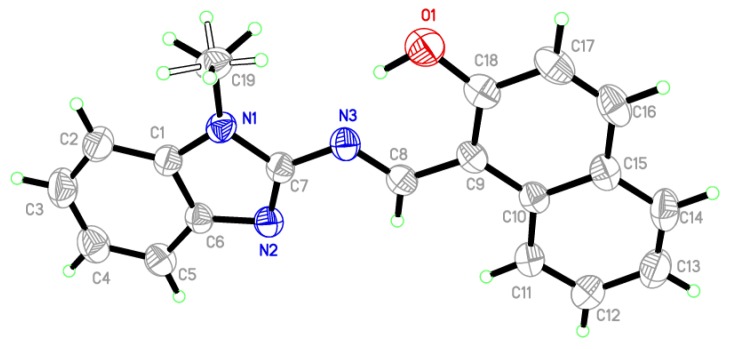
Ortep view of the single X-ray structure of the ligand at the 50% probability level.

Single crystal X-ray structural study revealed that ligand was crystallized in the monoclinic P21/c space group having the lattice parameters, a = 9.4568(6) Å, b = 12.9529(10) Å, c = 13.7156(8) Å, β = 116.171 (4) per unit cell. As shown in Figure 2, the asymmetric unit content for the ligand is consist of benzimidazole moiety linked to hydroxynaphthaldehyde moiety via imine bond (N3-O8 1.296 Å). All the atoms are in a single plane except the methyl hydrogens H19A, H19B, and H19C of benzimidazole moiety. Selected parameters are given in Table 1 and Table 2.

### 2.3. DNA Binding Studies

#### 2.3.1. Absorption Studies

**Figure 3:**
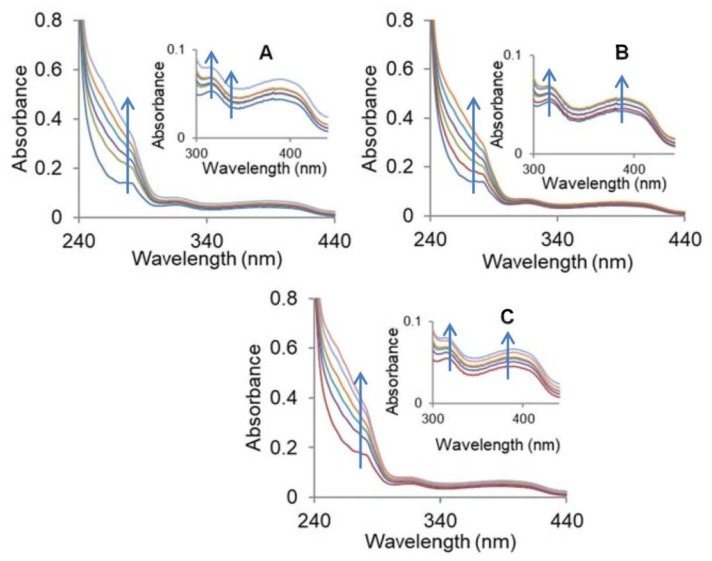
Absorption spectra of complexes (**A**) **1**, (**B**) **2**, and (**C**) **3**, with calf thymus-DNA (CT-DNA) in 5 mM Tris-HCl/50 mM NaCl buffer of pH 7.5 at room temperature.

Incremental addition of CT DNA to the metal complexes namely, copper (**1**), cobalt (**2**), and zinc (**3**) complexes exhibited hyperchromism of 35–38% along with a slight blue shift of ~2 nm. The results are suggestive of the non-intercalative binding mode of metal complexes **1**–**3** to DNA through electrostatic interactions at the surface and/or binding at one of the grooves, whereas partial intercalation possibilities cannot be ruled out (Figure 3) [28,29]. The extent of hyperchromism is generally linked to the strength of binding affinity of the complexes, and the order followed the pattern **1** > **2** > **3**. To quantify the affinity of the complexes with DNA, the Wolfe-Shimer equations [30] was used, and K_b_ (intrinsic binding constant) was calculated using the ratio of slope (1/ǀε_b_-ε_f_ǀ) to the intercept (1/K_b_/ǀε_b_-ε_f_ǀ) intercept. This corroborates well with the above order of hyperchromism and K_b_ values were found to be 6.65 × 10^4^ M^−1^ (**1**), 3.27 × 10^4^ M^−1^ (**2**), and 1.26 × 10^4^ M^−1^ (**3**), respectively. Thus, the results are evocative of the avid binding of the copper complex (**1**) as compared to the cobalt (**2**) and zinc (**3**) complexes. We found that the binding constants of metal complexes are lower as compared to that of the classical intercalator EtBr (K_b_ = 4.94 × 10^5^ M^−1^). The higher binding constant values of the complexes **1**–**3** are suggestive of the partial intercalative binding mode of the complexes **1**–**3** [31,32]. It is hypothesized that planar aromatic rings of the benzimidazole-derived ligand intercalate into the stacked bases of DNA.

#### 2.3.2. Fluorescence Quenching Experiments

**Figure 4:**
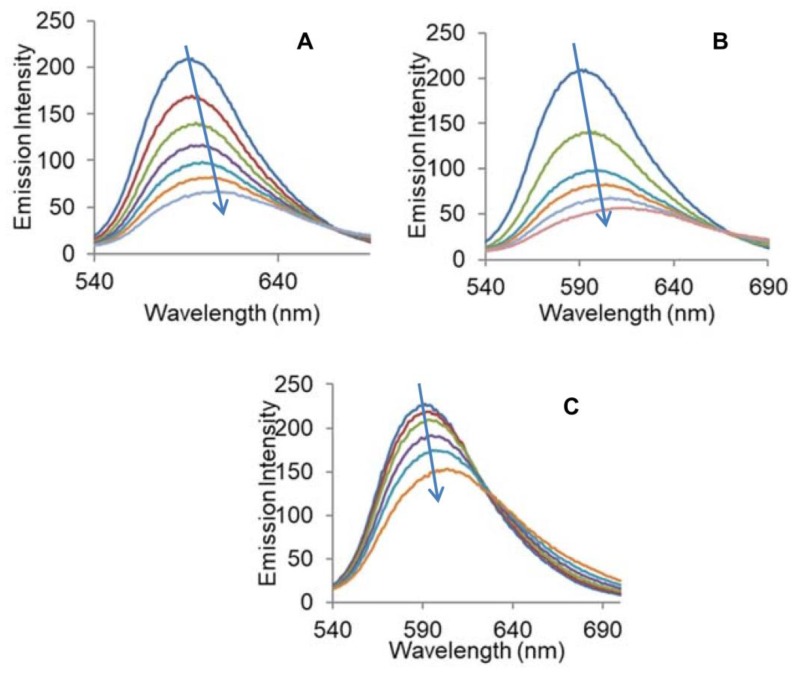
Quenching spectra of EtBr-DNA adduct by the complexes (**A) 1**, (**B**) **2**, and (**C**) **3**, with CT-DNA in 5 mM Tris-HCl/50 mM NaCl buffer of pH 7.5 at room temperature.

The results derived from the absorption studies prompted us to get an insight into the mechanism by which complexes **1**–**3** bind DNA. We performed EtBr displacement assay to examine the potential of complexes **1**–**3** to displace EtBr from EtBr-DNA complex. EtBr gives weak fluorescence emission alone, but when intercalated between the base pairs of DNA, it emits intense fluorescence. In this study, quenching by complexes **1**–**3** of EtBr-DNA adduct is analyzed, and the results are presented in Figure 4. The subsequent addition of complexes **1**–**3** quenches the fluorescence intensity of EtBr-DNA adduct along with redshift by replacing EtBr from the binding sites or through partial intercalation, making it difficult for EtBr to bind DNA. Thus, quenching in the fluorescence intensity of EtBr bound DNA in the presence of complexes **1**–**3** confirmed the partial intercalative mode of binding of the complexes. The following Stern-Volmer equations were used to deduce the extent of binding between complexes **1**–**3** and DNA.

F_o_/F = 1 + K_SV_ [Q] (1)

K_q_ = K_SV_/τ_o_ (2)

where, K_SV_ is Stern-Volmer quenching constant, K_q_ is biomolecular quenching constant, and τ_o_ is the lifetime of DNA fluorescence in the absence of any quencher. For DNA, τ_o_ is 10^−8^ s.

The Ksv values were found to be 5.02 × 10^4^ M^−1^, 3.10 × 10^4^ M^−1^, and 1.12 × 10^4^ M^−1^ for complexes **1**–**3**, respectively. Furthermore, apparent binding constant “K_app_” values were calculated using the equation

K_EthBr_ × [EtBr] = K_app_ × [complex] (3)

where, [complex] is the concentration at which the fluorescence intensity reduces to 50% of EtBr-DNA adduct and K_EtBr_ = 1 × 10^7^ M^−1^, [EtBr] = 6.5 mM. The apparent binding constant (K_app_) values were calculated to be 1.5 × 10^5^ M^−1^ and 2.5 × 10^6^ M^−1^ for ligand and complex **1**, respectively. We observed that K_app_ of complex **1** was 10-folds lower than the binding constant of classical intercalators and metallo-intercalators (10^7^ M^−1^).

Organic moiety (ligand) plays a pivotal role in the binding affinity towards biomolecules i.e., DNA. Thus, the designing of the ligand is also very important. Here, we have designed benzimidazole derived Schiff base with hydroxynaphthaldehyde. This biocompatible ligand with benzimidazole bioactive core possesses various characteristics like an aromatic ring which provides planarity to the molecule which is very important for the intercalative mode of binding, i.e., π-π* stacking between the adjacent base pair of DNA. However, the complexes **1**–**3** are not exactly planar because of the presence of N-CH_3_, so the typical intercalation is not possible due to steric hindrance, but partial intercalative mode is possible because of the presence of aromatic rings of naphthaldehyde can be envisioned in such complexes. This biocompatible ligand also facilitates the hydrogen bonding and electrostatic interaction with the DNA.

Although the skeleton (or ligand) of complexes **1**–**3** are same, they show different binding propensities towards DNA due to the presence of different central metal ions viz, Cu(II), Co(II), and Zn(II). The transition metal ions show a binding affinity towards purines nucleotide bases of DNA as they are more accessible in the major groove. A coordination bond between the central metal ion and N7 of the guanine bases has been reported [Ref]. Other possibilities such as a coordination bond between N7 of adenine or N3 of cytosine and thymine are also possible, though they are weak. Metal ions with a decrease in softness also have the potential to coordinate with the oxygen of the phosphate backbone of the DNA. Thus, the nature of central metal ions plays a major role in deciding coordination with a base or the phosphate oxygen. 

### 2.4. DNA Nuclease Activity and Mechanistic Pathways

**Figure 5:**
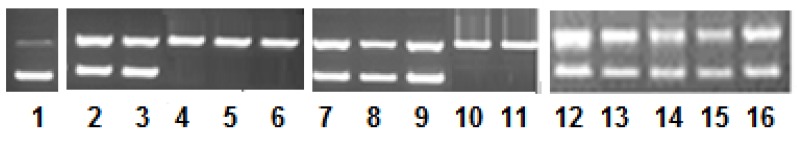
Concentration dependent pattern of pBR322 DNA (100 ng) by complex **1** (lane 2–6), complex **2** (lane 7–11), and complex **3** (lane 12–16) (0.5–2.5 μM), in 50 mM Tris-HCl/NaCl buffer pH 7.4 after 45 min of incubation. lane 1: DNA alone (control); lane 2: DNA + 0.5 μM **1**; lane 3: DNA + 1.0 μM **1**; lane 4: DNA + 1.5 μM **1**; lane 5: DNA + 2.0 μM **1**; lane 6: DNA + 2.5 μM **1**; lane 7: DNA + 0.5 μM **2**; lane 8: DNA + 1.0 μM **2**; lane 9: DNA + 1.5 μM **2**; lane 10: DNA + 2.0 μM **2**; lane 11: DNA + 2.5 μM; lane 12: DNA + 0.5 μM **3**; lane 13: DNA + 1.0 μM **3**; lane 14: DNA + 1.5 μM **3**; lane 15: DNA + 2.0 μM **3**; lane 16: DNA + 2.5 μM.

The nuclease activity of the complexes **1**–**3** was analyzed on pBR322 DNA using gel electrophoresis. Increasing concentration of complexes **1**–**3** (0.5–2.5 µM) was added to a fixed concentration of pBR322 DNA (100 µM) in 50 mM Tris-HCl/50 mM NaCl buffer of pH 7.4 and incubated for 45 min. The reaction mixture was subjected to 1% agarose gel electrophoresis at 30 V. The results exhibited concentration dependent pattern cleavage pattern (Figure 5). The electrophoretic results clearly showed the appearance of the open circular form (form II) of DNA from the native form (form I). It is interesting to note that there was no appearance of the linear circular form (form III) from the native form (form I). Thus, the single-stranded DNA cleavage was observed in all the three complexes. However, the nuclease activity of **1** was more prominent at lowers concentration as compared to complexes **2** and **3**. In the case of cobalt (**2**) and zinc (**3**) complexes, there is a clear indication of the hydrolysis of DNA. This is a typical characteristic of cobalt and zinc complexes to hydrolyse DNA [33,34,35,36].

**Figure 6:**
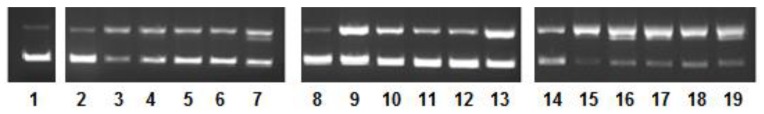
Electrophoretic pattern of pBR322DNA in the presence of scavengers/groove binders in 50 mM Tris-HCl/NaCl buffer pH 7.4 after 45 min. lane 1: DNA alone (control); lane 2: DNA + DAPI + **1**; lane 3: DNA + MG + **1**; lane 4: DNA + DMSO + **1**; lane 5: DNA + EtOH + **1**; lane 6: DNA + NaN_3_ + **1**; lane 7: DNA + SOD + **1**; lane 8: DNA + DAPI + **2**; lane 9: DNA + MG + **2**; lane 10: DNA + DMSO + **2**; lane 11: DNA + EtOH + **2**; lane 12: DNA + NaN_3_ + **2;** lane 13: DNA + SOD + **2**. lane 14: DNA + DAPI + **3**; lane 15: DNA + MG + **3**; lane 16: DNA + DMSO + **3**; lane 17: DNA + EtOH + **3**; lane 18: DNA + NaN_3_ + **3;** lane 19: DNA + SOD + **3**.

To study the mechanism of the cleavage of the DNA (Figure 6), the reaction was carried out in the presence of standard radical scavengers such as DMSO, EtOH (hydroxyl radical scavengers), NaN_3_ (singlet oxygen scavenger), and SOD (superoxide radical scavenger). In the case of copper (**1**) and cobalt (**2**) complexes, a free radical diffusion mechanism was observed, whereas zinc (**3**) complex ascertained hydrolytic cleavage mechanism as there is no inhibition of cleavage in the presence of scavengers. 

Furthermore, to get an insight into the binding site, the cleavage experiment was performed in the presence of groove binders such as methyl green (MG) as major groove binder and 4′,6-diamidino-2-phenylindole (DAPI) as minor groove binder (Figure 6). The results confirmed that copper complex (**1**) binds to the major groove, cobalt (**2**) complex exhibited affinity towards major as well as a minor groove, whereas zinc (**3**) complex does not display groove binding propensity.

### 2.5. In Vitro Cytotoxicity

#### 2.5.1. Analysis of Growth Inhibition Using MTT Assay

Considering the fact that synthesized drugs play a role as a potential anticancer agent in tumor growth suppression, the three novel complexes **1**, **2**, and **3** were investigated for their anticancer efficacy, in vitro. The cytotoxic effect of the synthesized complex was examined on a panel of cancer cells. The cytotoxic efficacy was expressed in terms of IC_50_ values, as shown in Table 3. In comparison with complexes **1** and **2**, complex **3** showed potential in vitro anticancer activity against all tested cancer cell lines namely HepG2 (Liver), SK-MEL-1 (Skin), HT018 (Colon) Hela (Cervical) and MDA-MB231 (Breast), in a dose-dependent manner. The tested cells after treatment, however, showed variability in growth inhibitions as calculated by the IC_50_ parameter. MDA-MB231 was observed to be the most sensitive to complex **3** followed by HeLa, SK-MEL-1, HepG2, and HT108. Further, no activity against selective cancer cell lines was observed with free ligands. Since, complex **3** was more potent as compared to complexes **1** and **2**, we continued our anticancer study further with complex **3** only. 

#### 2.5.2. Effect on Cancer Cell Adhesion and Cell Migration

**Figure 7:**
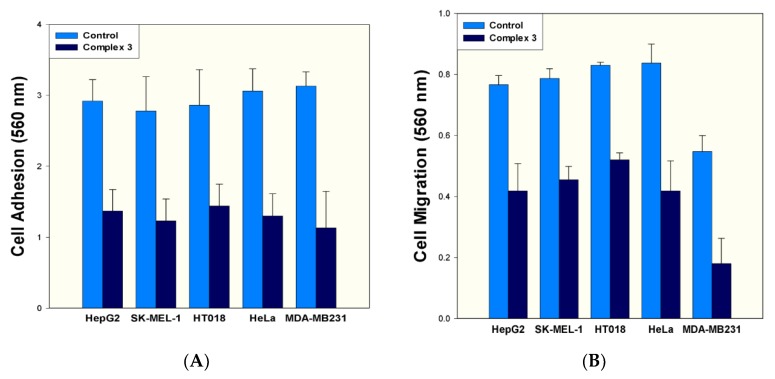
(**A**) Effect of complex **3** on cell adhesion against five cancer cell lines. Assays for adhesion and migration were performed with a cytoselect 24-well plate, and the absorbance of extracted samples was read at 560 nm. (**B**) Effect of complex **3** on cell migration against five cancer cell lines. Assays for adhesion and migration were performed with a cytoselect 24-well plate, and the absorbance of extracted samples was read at 560 nm.

Cancer metastasis is the spread of cancer cells to tissues and organs distinct from where the tumor was originated. Cancer cells are stuck together so that can move and migrate for malignancy. The properties of cell adhesion and cell migration are fundamental in regulating cell movement and cancer metastasis and are necessary for the cells to move into the bloodstream. Further characterization of anticancer activity of the complex **3** was conducted by adhesion and migration of cancer cells. The effect of complex **3** on cell adhesion was carried out at IC_50_ values on respective cell lines. Therefore, complex **3** was tested at the concentrations of 15.8 µM, 12.31 µM, 11.1 µM, 8.3 µM, 6.6 µM against HT018, HepG2, SK-MEL-1, HeLa, and MDA-MB231, respectively. We found that complex **3** inhibited cell adhesion by about 49.65–53.08% (Figure 7A). Moreover, the inhibitory effect of complex **3** was also determined on the metastatic property of cancer cell. The studies on cancer cell migration were also carried out considering the IC_50_ values used in cell migration assay. Therefore, complex **3** inhibited cell migration by about 37.34% (15.8 µM), 45.43% (12.31 µM), 42.18% (11.1 µM), 35.06% (8.3 µM), and 27.09% (6.6 µM) of HT 018, HepG2, SK-MEL-1, HeLa, and MDA-MB 231 respectively (Figure 7B). We, therefore, infer that the complex **3** was able to affect the metastatic potential of cancer cells by inhibiting their adhesion property. As far as migration is concerned, complex **3** inhibited migration phenomenon of tested cancer cells. However, the rate of inhibition varied with respective cell lines.

#### 2.5.3. Annexin V Apoptosis Detection Assay

**Figure 8:**
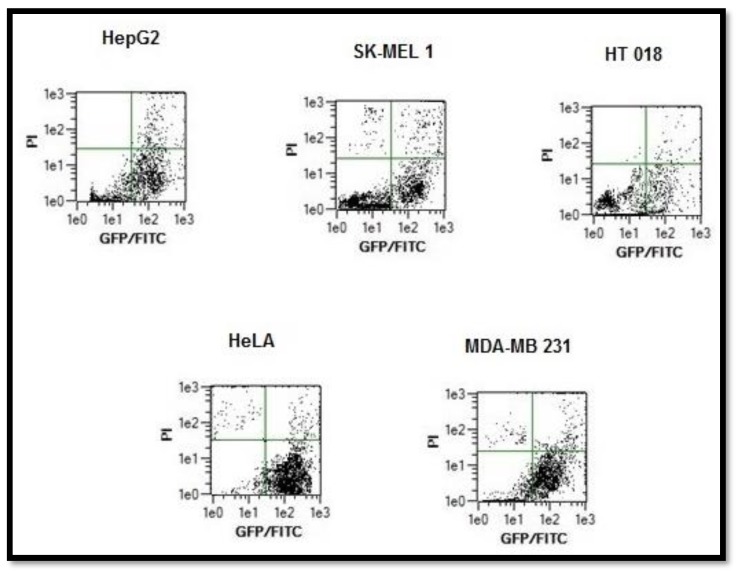
The apoptotic effect of complex **3** using Annexin-V staining of tested cancer cell lines. Dots represent cells as follows: lower left quadrant, normal cells (FITC^−^/PI^−^); lower right quadrant, early apoptotic cells (FITC^+^/PI^−^); upper left quadrant, necrotic cells (FITC^−^/PI^+^); upper right quadrant, late apoptotic cells (FITC^+^/PI^+^).

Apoptosis and necrosis are two major mechanisms of cell death. Cells that are damaged by external injury undergo necrosis, while cells that are induced to commit programmed suicide because of internal or external stimuli undergo apoptosis. An increasing number of chemo-preventive agents have been shown to stimulate apoptosis in pre-malignant and malignant cells in vitro or in vivo [37]. A gross majority of classical apoptotic attributes can be quantitatively assessed by flow cytometry. The apoptotic effect of complex **3** was evaluated using annexin-V staining. All the tested cancer cells were harvested after treatment by compound **3** for 48 h and incubated with annexin V-FITC and PI taking 10,000 cells as sample. The apoptotic effect of complex **3** was also analyzed on tested cancer cell lines using the respective IC_50_ concentrations. Figure 8 shows flow cytometric dot plots where annexin V-FITC binding is on the X-axis, and PI staining is on the Y-axis. Data of all cell lines after treatment with complex **3** showed a decrease in viable cancer cells. Distinct 22.3% (HepG2), 20.3% (SK-MEL-1), 14.1% (HT018), 34.5.2% (HeLa), and 45.2% (MDA-MB231) increase in apoptotic cell population was recorded. After 48 h exposure time, due to late apoptosis, the cell population increased to 11.8%, 13.0%, 1.5%, 9.2%, and 12.8% for HepG2, SK-MEL-1, HT018, HeLa, and MDA-MB231, respectively. Apoptosis was induced to some extent by complex **3**. We found that the compound **3** was most effective on MDA-MB- 231 cells.

### 2.6. In Vivo Toxicity of Complexes **1**–**3**

#### 2.6.1. Hematological Study

**Figure 9:**
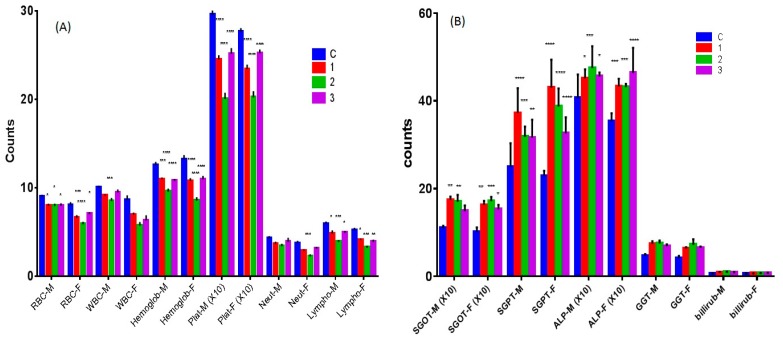

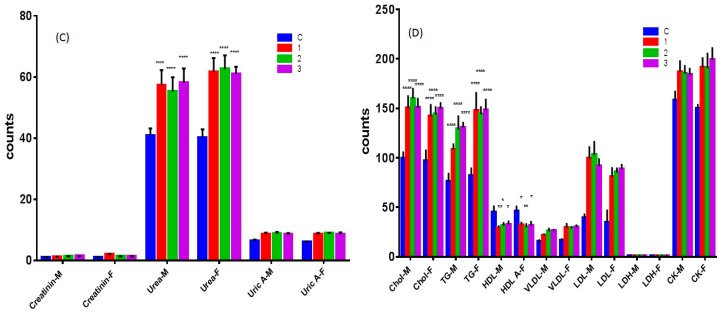
Chronic toxicity profile of complexes **1**, **2** and **3**. Effect of complexes **1**, **2** and **3** on (**A**) blood components, (**B**) biochemical parameters of liver function, (**C**) biochemical parameters of kidney function, and (**D**) biochemical parameters of lipids and heart function. M and F denote male and female mice, respectively. Statistical analysis was performed by two-way ANOVA, Dunett as post-Hoc test compared to control (**C**), *n* = 4. (* *p* < 0.05; ** *p* < 0.01; *** *p* < 0.005; **** *p* < 0.001).

The complexes **1** and **2** caused a decrease in RBC, WBC, hemoglobin, platelets, neutrophils, and lymphocytes counts (Figure 9A) significantly as compared to the control. However, complex **2** showed stronger activity than complex **1**. Complex **3** demonstrated a significant decrease in the counts of all the cells except WBC and neutrophils count in the male mice.

#### 2.6.2. Effect of Complexes **1**–**3** on Liver Function Tests

All test compounds increased the level of liver function parameters (SGOT, SGPT, GGT, and ALP) in both male and female mice significantly. However, complex **2** tended to be more potent than both the remaining complexes **1** and **3** on SGOT. Compound **1** seemed to be most potent among all the compounds (Figure 9B).

#### 2.6.3. Nephrotoxicity Tests of Complexes **1**–**3**

All the compounds increased urea levels in both male and female mice significantly. However, they did not affect creatinine and uric acid levels (Figure 9C).

#### 2.6.4. Effect of Complexes **1**–**3** on Lipid and Heart Function

All compounds increased triglycerides, cholesterol, LDL, VLDL levels but decreased HDL significantly in both male and female mice (Figure 9D). Moreover, all complexes significantly increased creatine kinase levels in both male and female mice. This lipid profile was not clinically favorable. Hence, the complexes are not advisable to the patients with high blood lipid levels and heart problems.

All the three compounds showed mild toxicity profile against blood cells, with compound **1** having the highest toxic activity. Despite the bone marrow suppression effect by all the three compounds, they were found much less aggressive than the reported toxicity of other metal complexes like platinum complexes [38]. All the complexes produced mild hepatotoxicity that was not obstructive as evidenced by unaltered bilirubin level which is known the hallmark of similar anticancer complexes [39]. Besides, the complexes didn’t perturb all the liver function indices which are quite indicative of mild toxicity. Moreover, the renal functions tests were also not affected except a slight increase in urea level. Additionally, cardiac enzymes were not affected as well (except CK). Nevertheless, it is important to note that the complexes increased cholesterol, triglycerides, and LDL. Hence, in a nutshell, in vivo toxicity study showed mild hematological and liver toxicity after chronic treatment with the complexes. However, the complexes are not clinically suitable for the patients suffering from hyperlipidemia and cardiac problems.

## 3. Experimental Section

### 3.1. Materials and Methods

Rosewell Park Memorial Institute (RPMI)-1640 medium, Dulbecco’s Minimum Essential Medium (DMEM), Fetal calf serum (FCS), dimethyl sulphoxide (DMSO), trypsin-EDTA solution, dithiothreitol (DTT); 3-4,5-dimethylthiazol-2-yl-2,5-diphenyltetrazolium bromide (MTT), and cisplatin were procured from Sigma-Aldrich (St. Louis, MO, USA). All other chemicals used were also of the highest purity. Cell adhesion and cell migration kits were obtained from Cell Biolabs, Inc. Vybrant Apoptosis Assay Kit was procured from Molecular Probe (Eugene, OR, USA).

### 3.2. Data Collection

Bruker APEX2; cell refinement: Bruker SAINT; data reduction: Bruker SAINT; program(s) used to solve structure: SHELXS97 (Sheldrick 2008); program(s) used to refine structure: SHELXL2014 (Sheldrick 2014); molecular graphics: Bruker SHELXTL; software used to prepare material for publication: Bruker SHELXTL. Absorption titrations were performed by maintaining a constant concentration of the complex while varying the nucleic acid (DNA) concentration. After equilibration for 5 min, the absorbance was recorded in the 350–600 nm range at room temperature. The titration was terminated when the wavelength and intensity of the Soret and Q bands for porphyrins did not change any more upon three successive additions of CT-DNA. Ethidium bromide (EtBr) displacement experiments were carried out by viewing the changes of the fluorescence intensity after excitation (λ_ex_) at 537 nm, and the emission spectrum was recorded in 540–690 nm range. A different aliquot of complexes **1**–**3** was added to an aqueous solution of EtBr-DNA system (DNA was pre-treated with EtBr in the ratio [DNA/EtBr] = 1 for 30 min at 25 °C).

### 3.3. Synthesis of Ligand “Bimnap”

The synthetic procedure adopted for the Schiff base was standard method, using EtOH as a solvent and the amine and aldehyde were mixed in 1:1 ratio and collected as yellow precipitate, washed with hexane, ether and dried in vacuum. Ligand “bimnap” was recrystallized in methanol to get a suitable single crystal for X-ray crystallography. 

Yield: (85%). m.p. 202 °C. Anal. Calc. for C_19_H_15_N_3_O: C, 75.73; H, 5.02; N, 13.94. Found: C, 75.76; H, 5.02; N, 13.95%. FT IR (cm^−1^): 3435, 3055, 1622, 1601, 1567, 1481, 1328, 826, 747. ^1^H-NMR (CDCl_3_, δ, 293 K): 10.36 (s, 1H, HC=N), 7.51–7.16 (ArH, 10H), 3.84 (s, 3H, N-CH_3_), 3.56 (br, s, 1H, OH). ESI-MS: 302.3.

### 3.4. Synthetic Procedure

The complexes were synthesized by adopting standard protocol using methanol as solvent and Cu(CH_3_COOH)_2_·*n*H_2_O, Co(CH_3_COOH)_2_·*n*H_2_O and Zn(CH_3_COOH)_2_·*n*H_2_O metal salts with Schiff base 1 in 1:2 ratios, respectively. The complexes were isolated as green, brown, and yellow precipitate, isolated, washed with ether, and hexane. 

#### 3.4.1. Copper Complex (**1**)

Yield: (82%). m.p. 245 °C. Anal. Calc. for C_38_H_28_N_6_O_2_Cu: C, 68.71; H, 4.25; N, 12.65. Found: C, 68.46; H, 4.23; N, 12.54%. FT-IR (cm^−1^): 3418, 3053, 1618, 1604, 1557, 1466, 1386, 1331, 835, 744, 673, 567, 463. ESI-MS: 665.1. Λ_M_ (1 × 10^−3^ M, DMSO): 10.03 Ω^−1^·cm^2^·mol^−1^ (non-electrolyte in nature). UV-vis (1 × 10^−3^ M, DMSO, λ_nm_): 660 nm. µ_eff_ (B.M.) = 1.93. EPR (solid state, g_av,_ 298 K) = 2.07. (see Figure S6)

#### 3.4.2. Cobalt Complex (**2**)

Yield: (74%). m.p. 296 °C. Anal. Calc. for C_38_H_28_N_6_O_2_Co: C, 69.19; H, 4.28; N, 12.74. Found: C, 68.91; H, 4.23; N, 12.70%. FT-IR (cm^−1^): 3398, 3051, 1618, 1605, 1571, 1483, 1376, 829, 741, 679, 557, 448. ESI-MS: 661.2. Λ_M_ (1 × 10^−3^ M, DMSO): 16.30 Ω^−1^·cm^2^·mol^−1^ (non-electrolyte in nature). UV-vis (1 × 10^−3^ M, DMSO, λ_nm_): 597 nm. µ_eff_ (B.M.) = 3.94.

#### 3.4.3. Zinc Complex (**3**)

Yield: (69%). m.p.195 °C. Anal. Calc. for C_38_H_28_N_6_O_2_Zn: C, 68.52; H, 4.24; N, 12.62. Found: C, 68.20; H, 4.23; N, 12.55%. FT IR (cm^−1^): 3396, 3049, 1615, 1603, 1567, 1476, 1368, 833, 745, 676, 555, 451. ^1^H-NMR (DMSO, δ, 293 K): 9.69 (s, 2H, HC=N), 8.69–6.56 (ArH, 20H), 1.90 (s, 6H, N-CH_3_). ESI-MS: 668.7.

### 3.5. DNA Binding and Nuclease Activity

DNA binding studies, ethidium bromide assay, and nuclease activity were performed by using standard protocols with some modification as stated by some of us previously (for experimental details, see the SI) [28,29,31,34,40,41].

### 3.6. Cell Lines and Culture Conditions

Human cancer cell lines; HepG2 (Liver), SK-MEL-1 (Skin), HT 018 (Colon), HeLa (Cervical) and MDA-MB231 (Breast) were procured from American Type Culture Collection (Rockville, MD). MDA-MB-231 and HepG2 cell lines were maintained in DMEM whereas, SK-MEL-1, HeLa and HT-018 were maintained in RPMI medium. The complete growth medium was supplemented with 10% (*v*/*v*) heat-inactivated FCS, 2 mM l-glutamine, antimycotic-antibiotic solution. All cells were maintained in a standard culture condition of 37 °C temperature and 95% humidified atmosphere containing 5% CO_2_. Cells were screened periodically for mycoplasma contamination.

### 3.7. MTT Assay

The novel complexes **1**–**3** were examined for their cytotoxicity against five different types of cancer cell lines viz., HepG2, SK-MEL-1, HT 018, HeLa and MDA-MB231 using a standard MTT reduction assay according to Khan et al. [42]. Briefly, cells at a concentration of 1 × 10^6^ cells/200 mL/well were plated in 96-well plates and further grown in their respective medium containing 10% FCS. After 24 h, cells were incubated with 0–25 µM concentrations of test compounds or respective free ligands. Cells with 0.1% DMSO (vehicle control) and cisplatin (positive control) were also cultured separately using the same conditions. Cells only were used as negative control. Ensuing 48 h incubation, old medium from treated cells was replaced with fresh medium. Now, MTT reagent (5 mg/mL in PBS) was added to each well and cells were further incubated for 2–3 h at 37 °C. After treatment, the supernatants were carefully removed, and 100 µL of DMSO was added to each well. Absorbance was measured at 620 nm in a multi-well plate reader, and drug sensitivity was expressed in terms of the concentration of drug required fora 50% reduction of cell viability (IC_50_).

### 3.8. Measurement of Cancer Cell Adhesion

Assay for adhesion was performed with cytoselect 24-well cell adhesion as per protocol [43]. For quantitative analysis, the IC_50_ concentration of respective complexes (in triplicate) was tested on HepG2, SK-MEL-1, HT 018, HeLa and MDA-MB231 cancer cells. Cancer cells (0.1–1.0 × 10^6^ cells/mL) cell suspension in serum-free medium was added to the inside of each well of a pre-warmed adhesion plate. The plates were incubated for 30–90 min in a CO_2_ incubator and treated as per the protocol. After air-drying the wells, stained adhered cells were extracted using extraction solution. Then the absorbance of the extracted sample (adhered cells) was read at 560 nm in a microtiter plate reader.

### 3.9. Measurement of Cancer Cell Migration

The cell migration assay was performed with a cytoselect 24-well cell migration assay according to the protocol [43] on HepG2, SK-MEL-1, HT 018, HeLa and MDA-MB231 cancer cell lines. For quantitative analysis, the IC_50_ concentration of respective complexes (in triplicate) was tested on HepG2 (Liver), SK-MEL-1 (Skin), HT018 (Colon) Hela (Cervical), and MDA-MB231 (Breast) cancer cells. The test compounds were supplemented with medium containing 10% fetal bovine serum in the lower well of the migration plate. To the inside of each insert, 100 µL of 0.5–1.0 × 10^6^ cells/mL of cell suspension was added. The plates were then incubated for 8 h at 37 °C in a humidified CO_2_ incubator. Finally, the absorbance of 100 µL of each sample was then read at 560 nm.

### 3.10. Analysis of Annexin-V Binding by Flow Cytometry

Annexin-V staining was performed according to the protocol [44]. For quantitative analysis, the IC_50_ concentration of complexes (in triplicate) was tested on HepG2 (Liver), SK-MEL-1 (Skin), HT018 (Colon) Hela (Cervical) and MDA-MB231 (Breast) cancer cells. Cancer cell (0.1–1.0 × 10^7^ cells/mL) suspension in serum-free medium was incubated with the respective compound in 6-well plates in a CO_2_ incubator. After treating with compound for 48 h, the cancer cells were harvested and incubated with annexin V-FITC and PI. The fluorescence emission of Annexin-V stained cells was measured at 530–575 nm in a flow cytometer (MACS Quant, Germany). Dots represent cells as follows: lower left quadrant, normal cells (FITC^−^/PI^−^); lower right quadrant, early apoptotic cells (FITC^+^/PI^−^); upper left quadrant, necrotic cells (FITC^−^/PI^+^); upper right quadrant, late apoptotic cells (FITC^+^/PI^+^).

### 3.11. In Vivo Toxicity

#### 3.11.1. Toxicity Study Design

Mice (total number = 20; weight average 25 gm; 10 male/10 female) were randomly divided into different groups (*n* = 6−10). Different doses of complexes **1** and **2** (0.5, 1, 2, 5, 8, and 10 g/kg) were administered intraperitoneally (one time). The complexes were suspended in 0.2% aqueous Tween 80 or 0.25% carboxymethylcellulose. The animals were observed for 72 h for signs of toxicity and mortality after administration of the drugs. LD_50_ was calculated according to a reliable method [45]. All the animal-based experiments were performed in compliance with the relevant laws and institutional guidelines and have been approved and permitted by the ethics committee (Letter No. CBR 4537) of the College of Pharmacy, King Saud University, Riyadh, KSA.

#### 3.11.2. Chronic Toxicity Study

A total of 40 male and 40 female Swiss albino mice were randomly allocated to the control and test groups (*n* = 10). The complexes **1** and **2** in each case were mixed with drinking water for the feasibility of administration due to long treatment duration. The dose selected was 1/10th of the LD_50_. The treatment was continued for 12 weeks (WHO Scientific Group, 1967) [46]. The animals were then observed for all physical symptoms of toxicity, body weight changes and mortality. One group of treated female rats were allowed to mate with the treated males, and thus the pregnant mice were studied. Their urine samples were collected 1–2 days before the end of the treatment. The treated animals were put on fasting for 12 h and then anesthetized with ether. Following, their blood samples were collected via heart puncture and centrifuged at 3000 rpm for 10 min. The plasma was then stored at −20 °C pending for analysis of the biochemical parameters. Vital organs were removed, weighed and investigated for apparent signs of toxicity and stored in 10% formalin for histological studies. The percentage of each organ relative to the body weight of the animal was also calculated.

#### 3.11.3. Hematological Studies

Whole blood samples from the treated animals were used for determination of some prominent hematological parameters. The samples were analyzed for WBC and RBC count, hemoglobin, platelets, neutrophils and lymphocytes measurement using Contraves Digicell 3100H (Zurich, Switzerland) [47].

#### 3.11.4. Serum Analysis of Biochemical Parameters

A colorimetric method was used for the determination of the biochemical parameters for liver function tests (SGPT, SGOT, GGT, ALP, bilirubin, and lipid profile) in plasma. The enzyme activity was quantified spectrophotometrically using commercial enzymatic kits (Crescent diagnostics test kits, SA).

### 3.12. Statistical Analysis

The results were presented as a mean ± standard error of the mean (S.E.M). Statistical differences were analyzed using ANOVA with Dunnett as a post-Hoc test. A value of *p* < 0.05 was considered statistically significant [48].

## 4. Conclusions

In this article, we have designed, synthesized and characterized three transition metal complexes, Cu(**1**), Co(**2**), and Zn(**3**) of biologically significant benzimidazole derived organic moiety. All the three complexes **1**–**3** were subjected to study binding with DNA as an essential feature of the metal-based anticancer chemotherapeutic. The results exhibited significant binding affinity of the complexes with DNA. Complex **1** exhibited the most avid binding as compared to **2** and **3**. Furthermore, the nuclease activity of the complexes **1**–**3** was studied on pBR322 DNA and their mechanistic pathways was elucidated in the presence of radical scavengers. The results showed significant nuclease activity at a relatively lower concentration of the complexes. Moreover, these complexes **1**–**3** were tested against 5 different human cancer cell lines, i.e., HepG2 (Liver), SK-MEL-1 (Skin), HT018 (Colon) Hela (Cervical), and MDA-MB231 (Breast) cancer cells. The results showed that the complex **3** was more active than complexes **1** and **2** contrasting with the above DNA binding results. Further, one of the fascinating outcomes of the present work is that the zinc complex (**3**) exhibited significant cytotoxicity as compared to cisplatin on all the tested cell lines. Also, the phenomenon of cell adhesion and migration was examined as an essential parameter of the death of cancer cells. Later, a vital prospect of toxicity was examined and studied the effect of complexes **1**–**3** on all the major organs of the mice. The results of chronic and acute toxicity ascertained that the degree of toxicity of all these potential drug candidates was very low. However, the present findings warrant more in vivo confirmatory experiments before judging the molecules as possible drug candidates.

## Figures and Tables

**Table 1:** Crystal data of the ligand “bimnap”.

Parameters	Ligand (bimnap)
Formula	C_19_H_15_N_3_O
Fw (g mol^–1^)	301.34
crystal system	Monoclinic
space group	P21/c
a (Å)	9.4568(6)
b (Å)	12.9529(10)
c (Å)	13.7156(8)
β (deg)	116.171(4)
U (Å^3^)	1507.83(18)
Z	4
µ (mm^–1^)	0.085
D (g cm^−3^)	1.327
F(000)	632
Temp (K)	293(2)
measured reflns	21459
unique reflns	4181
Ɵ range of data (°)	2.28–21.53
Goodness of fit on *F^2^*	1.035
Final *R*^b^ indices	*R*1 = 0.053
[*I* > 2σ(*I*)]	w*R*2 = 0.122
CCDC	1485309

**Table 2:** Selected bond angle (°) and bond length (Å).

	Bond Angle (°)		Bond Length (Å)	
C18-O1-H1O1	108.1 (18)		N3-C8-C9-C18	−1.5 (3)
N3-C8-C9-C10	179.09 (19)	C8-C9-C18-O1		−1.5 (3)
C19-N1-C7-N2	173.3 (2)		C19-N1-C1-C2	7.3 (4)
C1-N1-C7-N3	177.67 (17)		O1-C18	1.341 (3)
C8-N3-C7-N1	−178.96 (17)		O1-H1O1	0.97 (3)
N1-C19-H19B	112 (2)		N1-C7	1.368 (2)
N1-C19-H19A	107 (3)		N1-C1	1.380 (3)
O1-C18-C9	122.3 (2)		N1-C19	1.453 (3)
O1-C18-C17	116.6 (2)		N2-C7	1.316 (3)
N2-C7-N1	114.16 (19)		N2-C6	1.385 (3)
N2-C7-N3	128.24 (19)		N3-C8	1.296 (2)
N3-C8-C9	121.3 (2)		N1-C19-H19C	108 (2)

**Table 3:** IC_50_ values of complexes **1**–**3**, ligand, and cisplatin against five human cancer cell lines.

Sample Code	HepG2	SK-MEL-1	HT018	HeLa	MDA-MB 231
	(Liver) (µM)	(Skin) (µM)	(Colon) (µM)	(Cervical) (µM)	(Breast) (µM)
Complex **1**	26 ± 2.2 (Less potent)	54.7 ± 3.2 (Less potent)	29 ± 3.1 (Less potent)	23 ± 2.2	22 ± 2.4
Complex **2**	45 ± 2.6 (Less potent)	38 ± 2.3 (Less potent)	51.3 ± 2.3 (Less potent)	44.5 ± 2.2 (Less potent)	54.6 ± 2.6 (Less potent)
Complex **3**	12 ± 3.1	11.1 ± 2.3	15.8 ± 1.9	8.3 ± 1.5	6.66 ± 1.7
Ligand	NA	NA	NA	NA	NA
Vehicle control (0.1% DMSO)	NA	NA	NA	NA	NA
Cisplatin	6 ± 0.4	5.6 ± 0.8	5.7 ± 0.2	6 ± 0.6	3.1 ± 0.2

NA stands for No Activity.

## Supplementary Materials

The following are available online, Figures S1–S6: FTIR, NMR, EPR, Tables S1–S3: ligand X-ray crystallographic details.
