# Supplementary material for: Evaluation of Transition Metal Complexes of Benzimidazole-Derived Scaffold as Promising Anticancer Chemotherapeutics
Source: Molecules. 2018 May 21;23(5):1232. doi: 10.3390/molecules23051232 (PMC6100524; doi:10.3390/molecules23051232)
Supplement: Supplementary file 1 [file molecules-23-01232-s001.pdf]

Supporting Information

Evaluation of Transition Metal complexes of Benzimidazole-derived Scaffold as a Promising Anticancer Chemotherapeutics

Afzal Hussain,[a] Mohamed F. AlAjmi,[a] Md Tabish Rehman,[a] Azmat Ali Khan,[b] Pervez Alam Sheikh,[a] and Rais Ahmad Khan [c]*

[a]*Department of Pharmacognosy, College of Pharmacy, King Saud University, P.O. Box 2457, Riyadh 11451, Kingdom of Saudi Arabia.*

[b]*Department of Pharmaceutical Chemistry, College of Pharmacy, King Saud University, P.O. Box 2457, Riyadh 11451, Kingdom of Saudi Arabia.*

[c]*Department of Chemistry, College of Science, King Saud University, P.O. Box 2455, Riyadh 11451, Kingdom of Saudi Arabia.*

**Corresponding author*: raischem@gmail.com, Tel: +966 536745404.

Experimental Section

Absorption spectral titration experiments were performed with UV-1700 PharmaSpec UV-visible spectrophotometer (Shimadzu) spectrophotometer. UV-visible data were reported as λ_{max}/nm. Emission intensity measurements were carried out using Shimadzu RF-5301PC spectrofluorophotometer at room temperature. DNA binding experiments including absorption spectral traces, luminescence and nuclease activity were performed according to the standard methods [1] and practices previously adopted by our laboratory [2]. A solution of CT-DNA in the Tris-HCl buffer gave a ratio of UV absorbance at 260 and 280 nm of about 1.85 indicating that the DNA was sufficiently free of protein. The DNA concentration per nucleotide was determined by absorption spectroscopy using the molar absorption coefficient (6600 M^{-1} cm^{-1}) at 260 nm. Cleavage experiments were performed with the help of Axygen electrophoresis supported by Genei power supply with a potential range of 10–100 V, visualized and photographed by a Vilber-INFINITY Gel documentation system.

Absorption titration of metal complexes binding to DNA

The binding strength of complexes **1-3** with CT-DNA was determined by titrating different concentrations of the complexes **1-3** with a fixed amount of DNA. The binding constant (K_b) was determined using equation (1) and monitoring the changes in the absorbance of the π-π* and n- π bands with increasing concentration of CT-DNA.

$$[DNA]/\varepsilon_a-\varepsilon_f = [DNA]/\varepsilon_b-\varepsilon_f + 1/K_b(\varepsilon_b-\varepsilon_f) \quad (1)$$

where ε_a, ε_f, and ε_b correspond to (A_{obsd} /[M]), the extinction coefficient of free metal [Cu(II)/Co(II)/Zn(II)] complex and the extinction coefficient for the metal complex in the in the fully bound forms, respectively. A plot of [DNA]/ ε_a- ε_f vs [DNA], where [DNA]

is the concentration of DNA in the base pair, and K_b as the ratio of the slope to the intercept.

Ethidium Bromide (EtBr) displacement Assay

The molecular fluorophore EtBr emits intense fluorescence in the presence of CT DNA due to its strong intercalation between the adjacent DNA base pairs. Addition of the second molecule, which binds to DNA more strongly than EtBr, would quench the DNA-induced EhBr either by replacing the EtBr and/or by accepting the excited-state electron of the EthBr through a photoelectron transfer mechanism.

The extent of emission quenching of the EtBr bound to DNA would reflect the extent of DNA binding affinity of the complex. On addition of complex to CT DNA pre-treated with EtBr ([DNA]/[EtBr] = 1) a decrease in emission intensity was observed.

The quantitative quenching extent that is, Ksv was evaluated by following the Stern–Volmer equation:

$$Io/ I = 1 + Ksv\ [complex]$$

where Io and I are the emission intensities in the absence and the presence of the complex, respectively

Nuclease Activity

Concentration dependent

Cleavage experiments of supercoiled pBR322 DNA (100 ng) by the complexes **2** and **3** (0.5–2.5 μM) in 50 mM Tris-HCl/50 mM NaCl buffer at pH 7.4 were carried out by agarose gel electrophoresis. The samples were incubated for 45 min at 37 ℃. A loading buffer containing 25% bromophenol blue, 0.25% xylene cyanol, and 30% glycerol was

added, and electrophoresis was carried out at 30 V for 3 h in Tris-HCl buffer using 1% agarose gel containing 1.0 μgmL^{-1} ethidium bromide (EtBr).

Mechanistic pathways

A similar protocol was adopted for mechanistic pathway with the addition of radical scavengers. The cleavage experiments of pBR322 plasmid DNA (100 ng) by a fixed concentration of complexes **1-3** (1.5 μM) with different additives for an incubation time of 45 min at 37 ℃ was studied. Control; DNA alone, DMSO (5 μL), EtOH (5 μL), NaN_3 (20 mM), SOD (5 units), DAPI (8 mM), methyl green (2.5 μL of a 0.01 mg mL^{-1} solution).

References

1. (a) J. Marmur, J. Mol. Biol., 1961, 3, 208–218; (b) M. E. Reicmann, S. A. Rice, C. A. Thomas and P. Doty, J. Am. Chem. Soc., 1954, 76, 3047–3053; (c) A. Wolfe, G. H. Shimer and T. Meehan, Biochemistry, 1987, 26, 6392–6396; (d) J. R. Lakowicz and G. Weber, Biochemistry, 1973, 12, 4161–4170.

2. (a) S. Tabassum, S. Mathur, F. Arjmand, K. Mishra and K. Banerjee,Metallomics, 2012, 4, 205–217; (a) F. Arjmand, F. Sayeed, S .Parveen, S. Tabassum, A. S. Juvekar and S. M. Zingde,Dalton Trans., 2013, 42, 3390–3401; (b) S. Tabassum, W. M. Al-Asbahy, M. Afzal, F. Arjmand and V. Bagchi, Dalton Trans., 2012, 41,4955–4964; (c) S. Tabassum, M. Zaki, M. Afzal and F. Arjmand, Dalton Trans., 2013, 42, 10029–10041; (d) M. Chauhan, K. Banerjee and F. Arjmand, Inorg. Chem., 2007, 46,3072–3082; (e) R. A. Khan, A. Ahmad, R. Kakkar, D. Gupta, V. Bagchi and S. Tabassum,Organometallics, 2013, 32, 2546–2551.

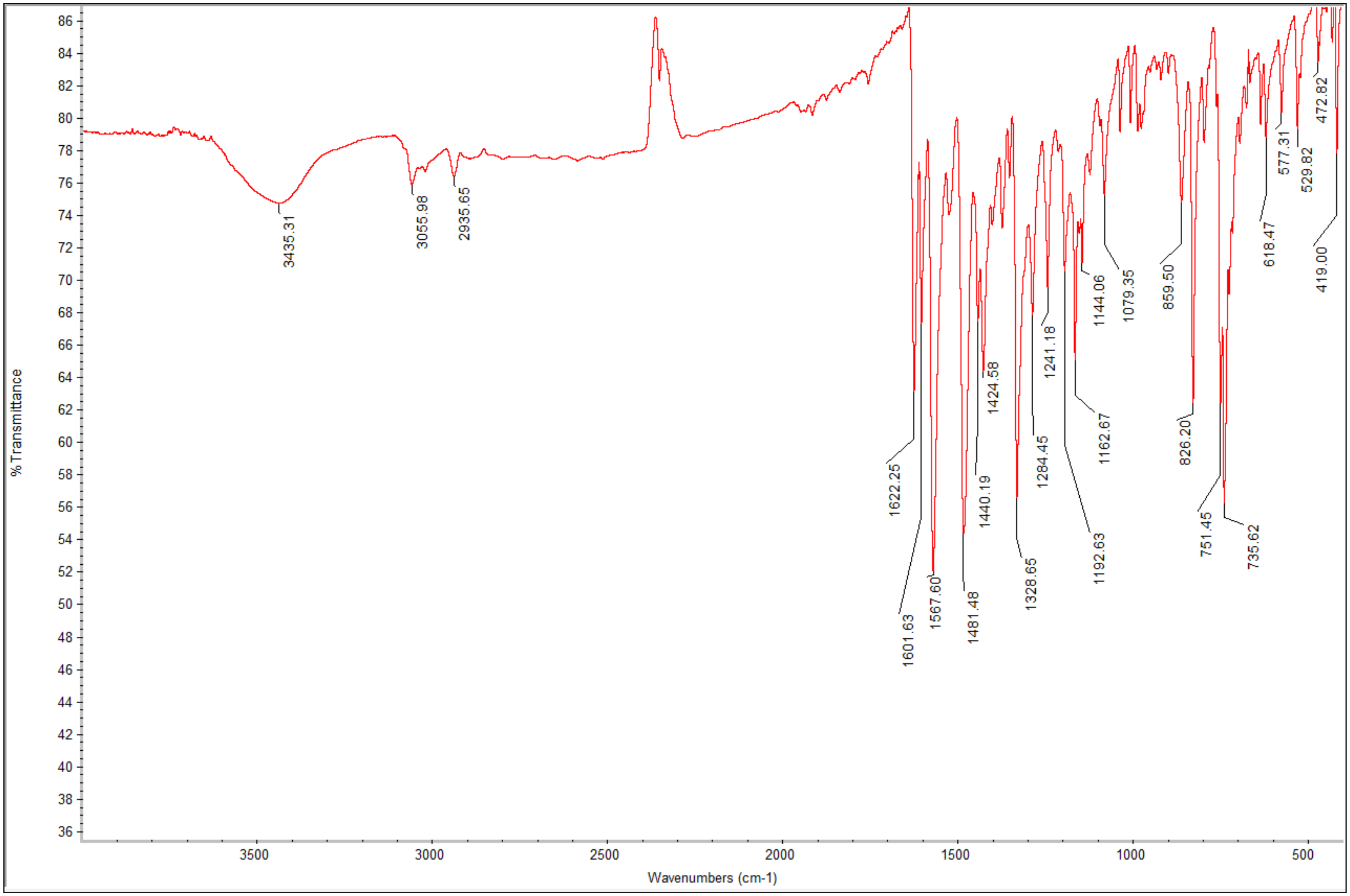

Figure S1: FT-IR spectrum of ligand.

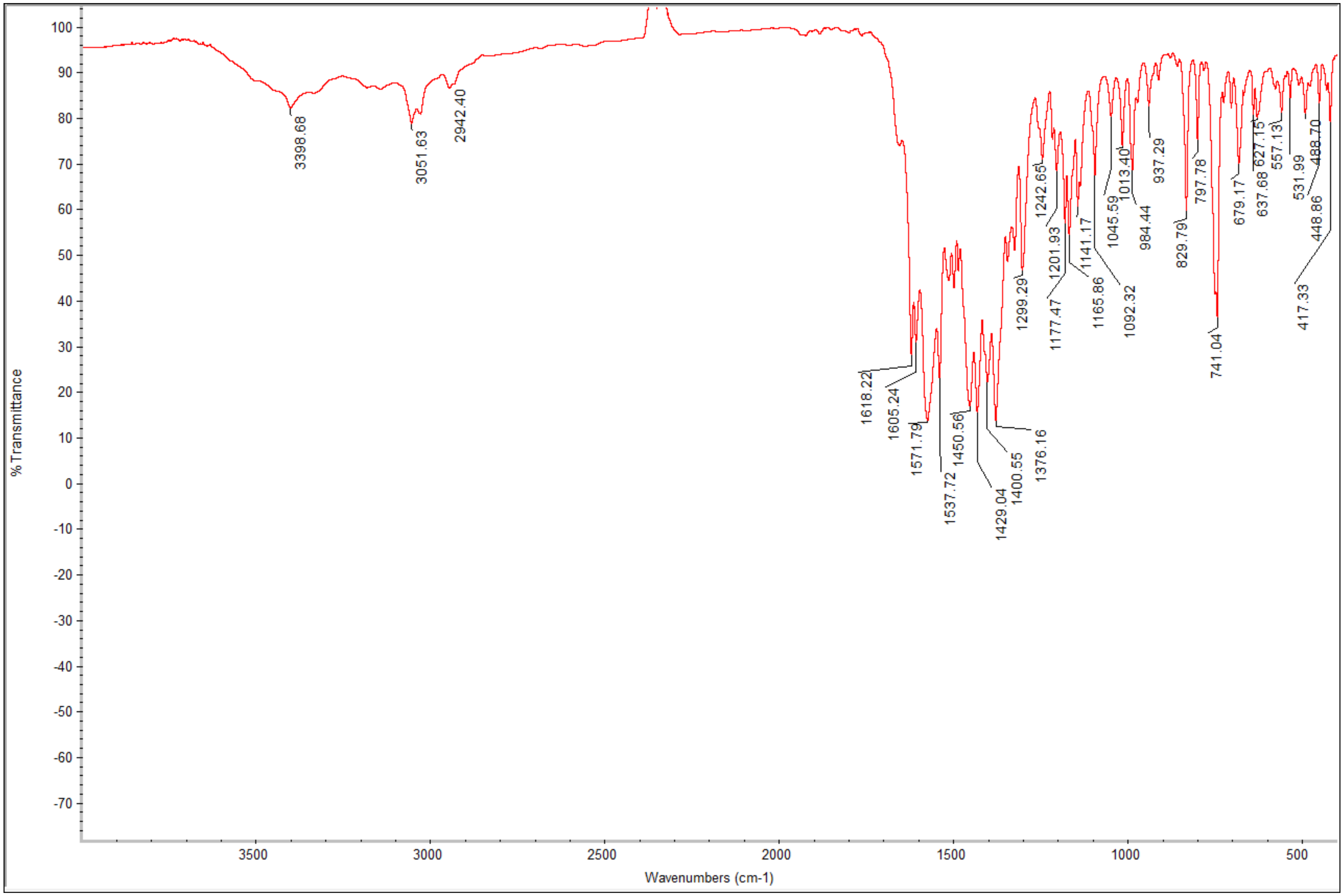

Figure S2: FT-IR spectrum of copper complex (**1**).

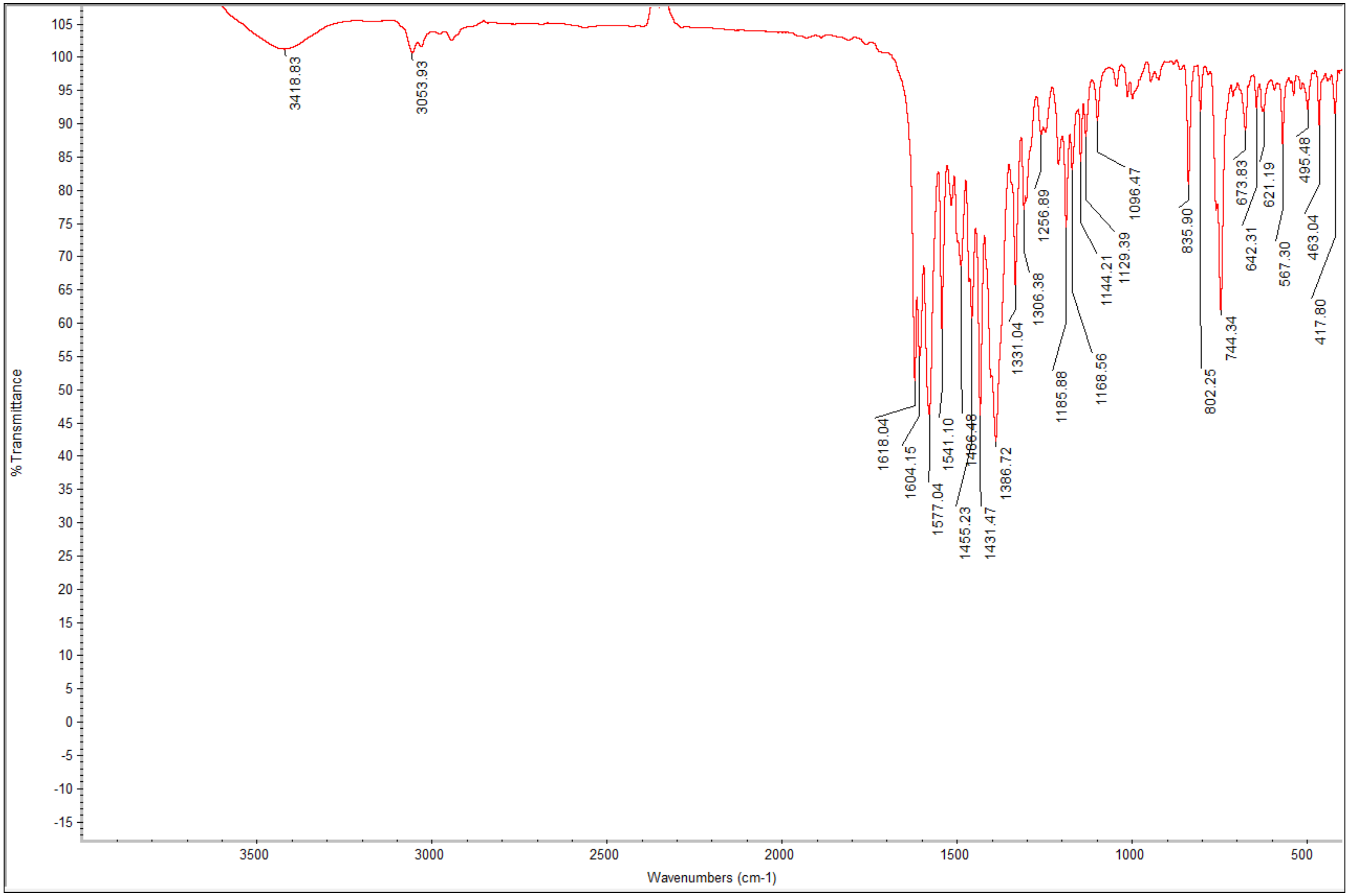

Figure S3: FT-IR spectrum of cobalt complex (**2**).

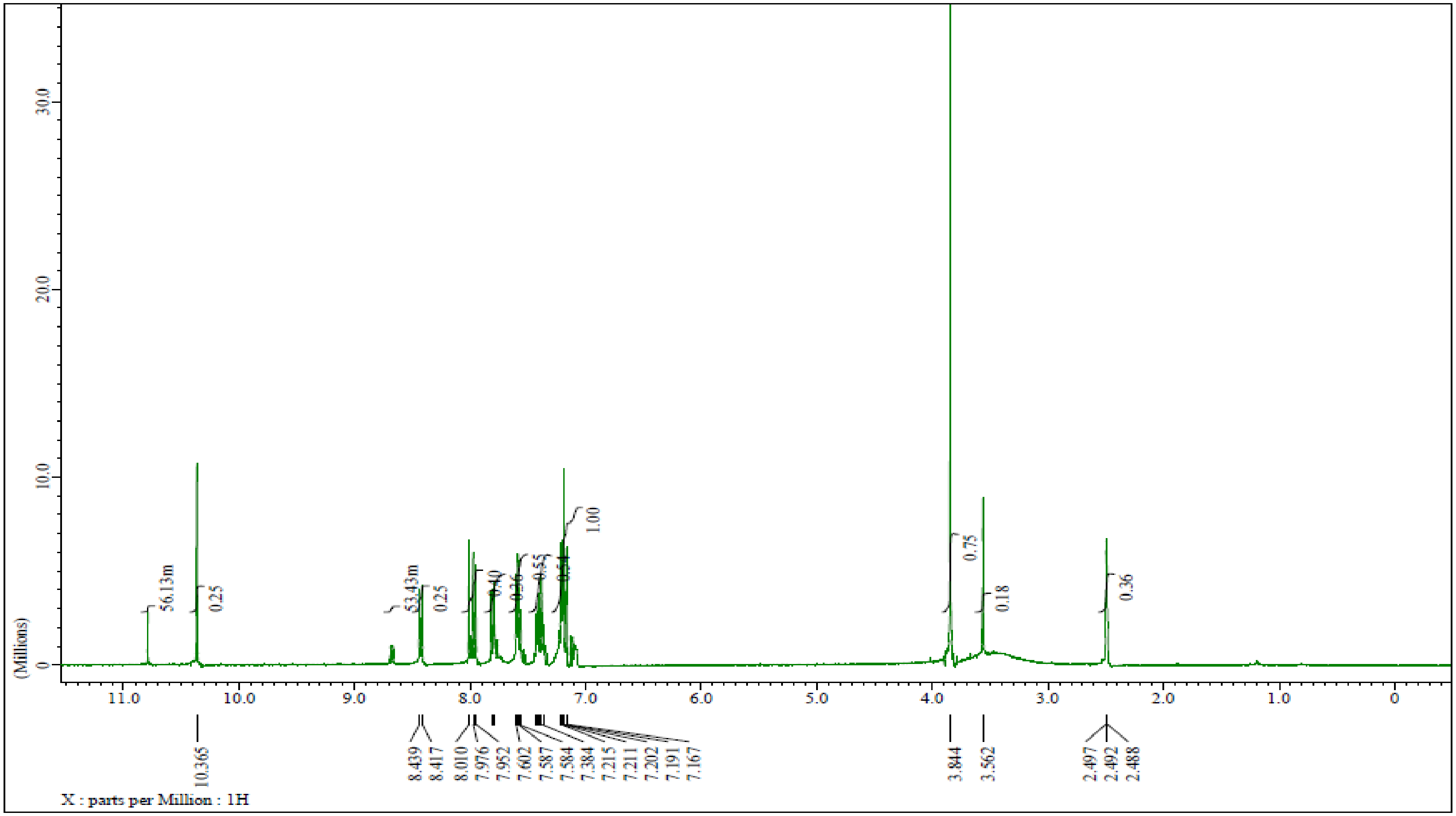

Figure S4: ^{1}H NMR spectrum of Schiff base ligand 'bimnap'**.**

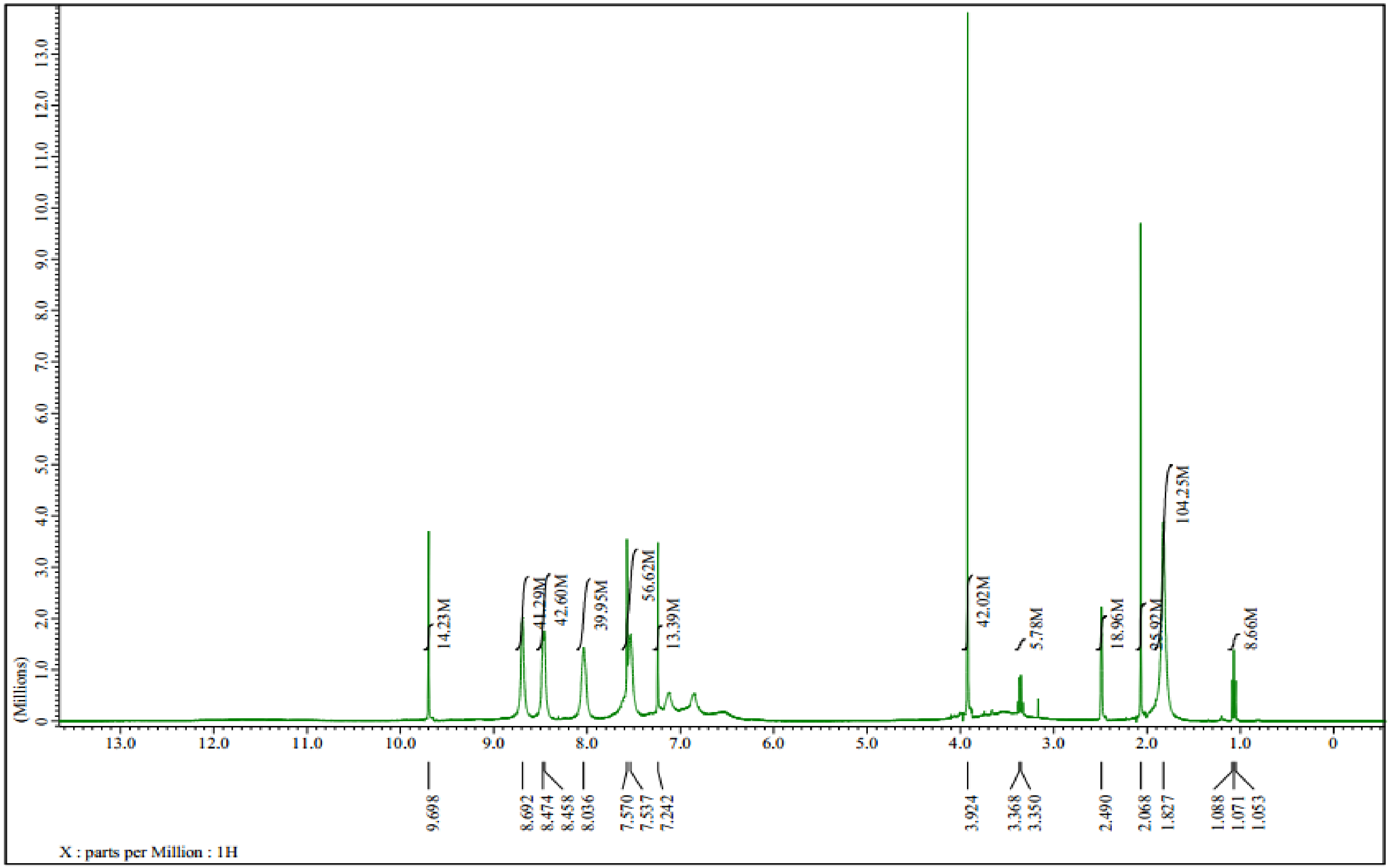

Figure S5: ^{1}H NMR spectrum of Zinc complex (**3**).

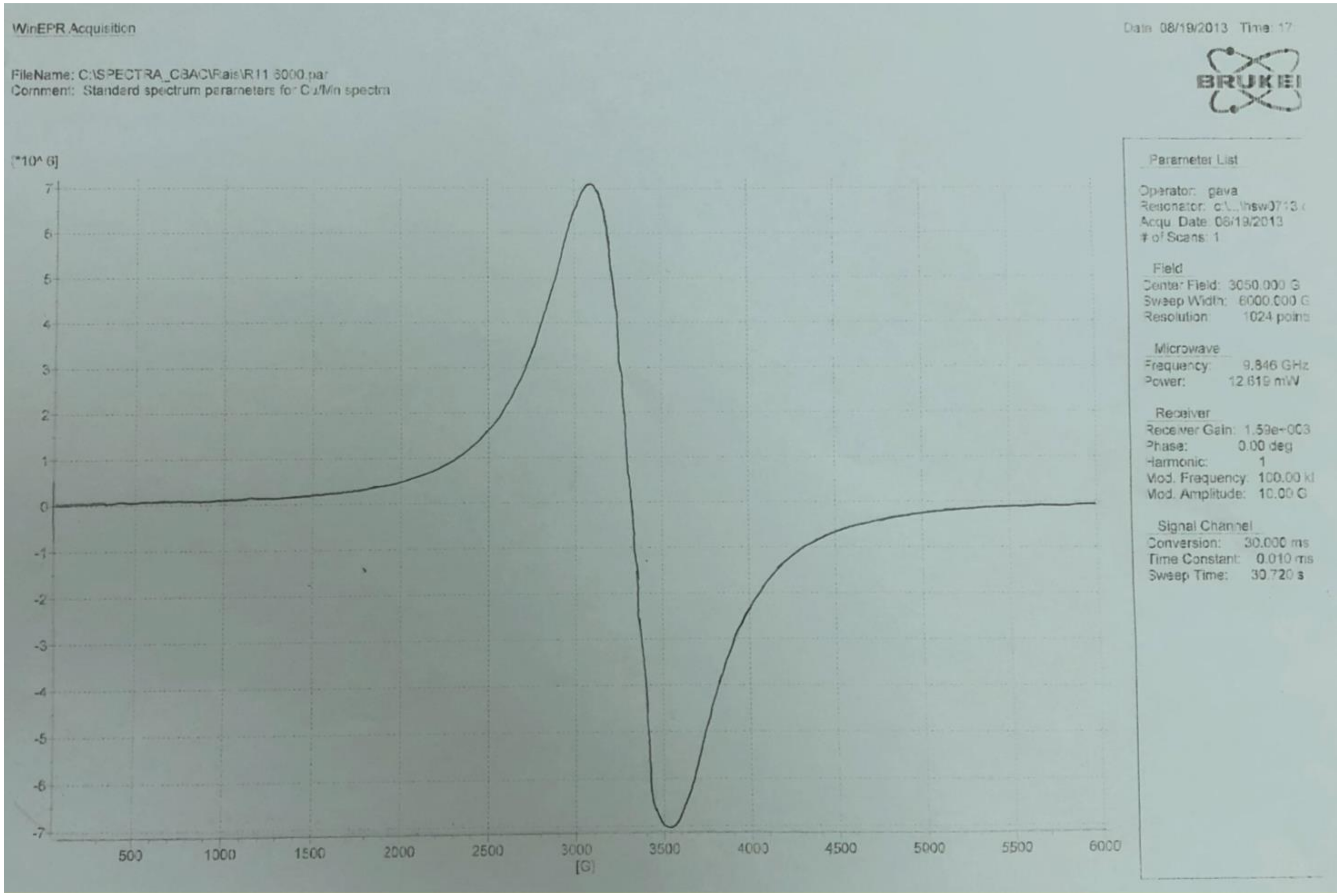

Figure S6: EPR spectrum of Copper complex (**1**).

Table S1. Fractional atomic coordinates and isotropic or equivalent isotropic displacement parameters (Å^2)

	x	***y***	***z***	U_{iso}*/U_{eq}
**O1**	−0.4570 (2)	0.82934 (13)	0.48852 (15)	0.0637 (5)
**N1**	−0.2071 (2)	1.02395 (13)	0.37061 (13)	0.0429 (5)
**N2**	−0.1071 (2)	1.12533 (14)	0.51934 (14)	0.0451 (5)
**N3**	−0.2708 (2)	0.97809 (13)	0.51270 (14)	0.0444 (5)
**C1**	−0.1242 (2)	1.10114 (17)	0.34909 (17)	0.0431 (5)
**C2**	−0.0992 (3)	1.1225 (2)	0.25908 (19)	0.0594 (7)
**H2A**	−0.1414	1.0810	0.1974	0.071*
**C3**	−0.0091 (3)	1.2078 (2)	0.2648 (2)	0.0685 (8)
**H3A**	0.0086	1.2251	0.2053	0.082*
**C4**	0.0561 (3)	1.2687 (2)	0.3579 (2)	0.0646 (7)
**H4A**	0.1179	1.3253	0.3598	0.078*
**C5**	0.0308 (3)	1.24694 (18)	0.4470 (2)	0.0540 (6)
**H5A**	0.0753	1.2878	0.5090	0.065*
**C6**	−0.0623 (2)	1.16272 (17)	0.44260 (17)	0.0421 (5)
**C7**	−0.1924 (2)	1.04330 (17)	0.47271 (17)	0.0404 (5)
**C8**	−0.2621 (2)	0.99451 (16)	0.60840 (17)	0.0428 (5)
**H8A**	−0.2020	1.0494	0.6496	0.051*
**C9**	−0.3434 (2)	0.92960 (16)	0.65204 (17)	0.0410 (5)
**C10**	−0.3321 (2)	0.94678 (16)	0.75904 (17)	0.0420 (6)
**C11**	−0.2426 (3)	1.02717 (18)	0.82826 (18)	0.0543 (6)
**H11A**	−0.1844	1.0707	0.8059	0.065*
**C12**	−0.2402 (3)	1.0421 (2)	0.92793 (19)	0.0636 (7)
**H12A**	−0.1801	1.0956	0.9721	0.076*
**C13**	−0.3257 (3)	0.9790 (2)	0.9640 (2)	0.0678 (7)
**H13A**	−0.3248	0.9909	1.0312	0.081*
**C14**	−0.4109 (3)	0.8994 (2)	0.9007 (2)	0.0625 (7)
**H14A**	−0.4666	0.8564	0.9256	0.075*
**C15**	−0.4162 (3)	0.88101 (17)	0.79830 (19)	0.0476 (6)
**C16**	−0.5071 (3)	0.79907 (18)	0.7308 (2)	0.0585 (7)
**H16A**	−0.5613	0.7553	0.7563	0.070*

**C17**	−0.5171 (3)	0.78298 (17)	0.6310 (2)	0.0578 (7)
**H17A**	−0.5772	0.7285	0.5890	0.069*
**C18**	−0.4368 (3)	0.84842 (17)	0.5900 (2)	0.0481 (6)
**C19**	−0.2795 (4)	0.9344 (2)	0.3032 (2)	0.0555 (7)
**H19A**	−0.299 (7)	0.952 (4)	0.229 (4)	0.083*
**H19B**	−0.208 (6)	0.871 (4)	0.329 (4)	0.083*
**H19C**	−0.391 (6)	0.927 (4)	0.298 (4)	0.083*
**H19Z**	−0.385 (7)	0.958 (4)	0.234 (5)	0.027 (16)*
**H19Y**	−0.194 (7)	0.908 (5)	0.276 (5)	0.05 (2)*
**H19X**	−0.300 (7)	0.880 (4)	0.351 (4)	0.031 (18)*
**H1O1**	−0.395 (4)	0.878 (2)	0.471 (2)	0.117 (12)*

Table S2. Atomic displacement parameters (Å^2)

	U^{11}	U^{22}	U^{33}	U^{12}	U^{13}	U^{23}
O1	0.0704 (12)	0.0566 (11)	0.0712 (13)	−0.0095 (9)	0.0375 (10)	−0.0206 (9)
N1	0.0430 (11)	0.0464 (11)	0.0402 (11)	0.0042 (9)	0.0193 (9)	−0.0039 (9)
N2	0.0434 (11)	0.0484 (12)	0.0460 (11)	0.0008 (9)	0.0220 (9)	−0.0022 (9)
N3	0.0433 (11)	0.0459 (11)	0.0491 (12)	0.0050 (9)	0.0250 (9)	−0.0005 (9)
C1	0.0388 (13)	0.0504 (14)	0.0409 (13)	0.0097 (11)	0.0184 (11)	0.0063 (11)
C2	0.0595 (16)	0.0752 (19)	0.0468 (14)	0.0050 (14)	0.0264 (13)	0.0006 (13)
C3	0.0686 (18)	0.087 (2)	0.0609 (18)	0.0086 (17)	0.0383 (15)	0.0214 (16)
C4	0.0628 (17)	0.0639 (17)	0.0728 (19)	0.0011 (14)	0.0350 (15)	0.0140 (15)
C5	0.0515 (15)	0.0521 (15)	0.0605 (16)	0.0014 (12)	0.0266 (13)	0.0020 (12)
C6	0.0386 (13)	0.0441 (13)	0.0462 (13)	0.0071 (11)	0.0211 (11)	0.0041 (11)
C7	0.0368 (13)	0.0442 (13)	0.0427 (13)	0.0091 (11)	0.0199 (11)	0.0035 (11)
C8	0.0387 (13)	0.0412 (13)	0.0492 (14)	0.0040 (10)	0.0200 (11)	0.0004 (11)
C9	0.0358 (12)	0.0369 (12)	0.0531 (14)	0.0055 (10)	0.0222 (11)	0.0015 (11)
C10	0.0365 (13)	0.0405 (13)	0.0503 (14)	0.0062 (10)	0.0202 (11)	0.0064 (11)
C11	0.0555 (16)	0.0589 (16)	0.0536 (15)	−0.0058 (13)	0.0287 (13)	0.0030 (12)
C12	0.0655 (17)	0.0737 (19)	0.0515 (16)	−0.0048 (14)	0.0257 (14)	−0.0023 (13)
C13	0.0769 (19)	0.079 (2)	0.0533 (16)	0.0036 (16)	0.0338 (15)	0.0097 (15)
C14	0.0674 (18)	0.0642 (18)	0.0680 (18)	0.0126 (15)	0.0409 (15)	0.0264 (15)
C15	0.0442 (14)	0.0428 (14)	0.0591 (15)	0.0108 (11)	0.0258 (12)	0.0152 (12)
C16	0.0546 (16)	0.0441 (15)	0.085 (2)	0.0046 (12)	0.0384 (15)	0.0161 (14)
C17	0.0521 (15)	0.0379 (14)	0.085 (2)	−0.0044 (11)	0.0312 (14)	−0.0041 (13)
C18	0.0432 (14)	0.0394 (13)	0.0635 (16)	0.0054 (11)	0.0251 (12)	−0.0040 (12)
C19	0.0543 (18)	0.0586 (17)	0.0492 (17)	0.0029 (14)	0.0189 (16)	−0.0104 (14)

Table S3. Geometric parameters (Å)

C7—N1—C1—C2	−179.0 (2)	N3—C8—C9—C18	−1.5 (3)
C19—N1—C1—C2	7.3 (4)	N3—C8—C9—C10	179.09 (19)
C7—N1—C1—C6	0.6 (2)	C18—C9—C10—C11	−179.2 (2)
C19—N1—C1—C6	−173.1 (2)	C8—C9—C10—C11	0.2 (3)
N1—C1—C2—C3	179.8 (2)	C18—C9—C10—C15	−0.1 (3)
C6—C1—C2—C3	0.2 (3)	C8—C9—C10—C15	179.31 (19)
C1—C2—C3—C4	1.1 (4)	C15—C10—C11—C12	−1.3 (3)
C2—C3—C4—C5	−1.0 (4)	C9—C10—C11—C12	177.8 (2)
C3—C4—C5—C6	−0.3 (4)	C10—C11—C12—C13	−0.1 (4)
C7—N2—C6—C5	−178.9 (2)	C11—C12—C13—C14	1.4 (4)
C7—N2—C6—C1	0.5 (2)	C12—C13—C14—C15	−1.1 (4)
C4—C5—C6—N2	−179.1 (2)	C13—C14—C15—C16	−178.9 (2)
C4—C5—C6—C1	1.6 (3)	C13—C14—C15—C10	−0.4 (3)
N1—C1—C6—N2	−0.7 (2)	C11—C10—C15—C14	1.6 (3)
C2—C1—C6—N2	178.95 (19)	C9—C10—C15—C14	−177.58 (19)
N1—C1—C6—C5	178.77 (18)	C11—C10—C15—C16	−179.82 (19)
C2—C1—C6—C5	−1.6 (3)	C9—C10—C15—C16	1.0 (3)
C6—N2—C7—N1	−0.1 (2)	C14—C15—C16—C17	177.7 (2)
C6—N2—C7—N3	−177.9 (2)	C10—C15—C16—C17	−0.8 (3)
C1—N1—C7—N2	−0.4 (2)	C15—C16—C17—C18	−0.3 (4)
C19—N1—C7—N2	173.3 (2)	C8—C9—C18—O1	−1.5 (3)
C1—N1—C7—N3	177.67 (17)	C10—C9—C18—O1	177.99 (19)
C19—N1—C7—N3	−8.6 (3)	C8—C9—C18—C17	179.6 (2)
C8—N3—C7—N2	−1.2 (3)	C10—C9—C18—C17	−1.0 (3)
C8—N3—C7—N1	−178.96 (17)	C16—C17—C18—O1	−177.8 (2)
C7—N3—C8—C9	179.14 (18)	C16—C17—C18—C9	1.2 (3)
O1—C18	1.341 (3)	C9—C10	1.441 (3)
O1—H1O1	0.97 (3)	C10—C11	1.412 (3)
N1—C7	1.368 (2)	C10—C15	1.424 (3)
N1—C1	1.380 (3)	C11—C12	1.371 (3)
N1—C19	1.453 (3)	C11—H11A	0.9300
N2—C7	1.316 (3)	C12—C13	1.387 (3)
N2—C6	1.385 (3)	C12—H12A	0.9300
N3—C8	1.296 (2)	C13—C14	1.359 (3)
N3—C7	1.387 (3)	C13—H13A	0.9300
C1—C2	1.383 (3)	C14—C15	1.403 (3)

C1—C6	1.401 (3)	C14—H14A	0.9300
C2—C3	1.377 (3)	C15—C16	1.421 (3)
C2—H2A	0.9300	C16—C17	1.345 (3)
C3—C4	1.392 (3)	C16—H16A	0.9300
C3—H3A	0.9300	C17—C18	1.411 (3)
C4—C5	1.374 (3)	C17—H17A	0.9300
C4—H4A	0.9300	C19—H19A	0.98 (5)
C5—C6	1.386 (3)	C19—H19B	1.03 (5)
C5—H5A	0.9300	C19—H19C	1.03 (5)
C8—C9	1.436 (3)	C19—H19Z	1.07 (6)
C8—H8A	0.9300	C19—H19Y	1.08 (7)
C9—C18	1.396 (3)	C19—H19X	1.03 (6)
C18—O1—H1O1	108.1 (18)	C12—C11—C10	121.1 (2)
C7—N1—C1	105.98 (17)	C12—C11—H11A	119.4
C7—N1—C19	127.2 (2)	C10—C11—H11A	119.4
C1—N1—C19	126.5 (2)	C11—C12—C13	121.1 (3)
C7—N2—C6	103.90 (17)	C11—C12—H12A	119.4
C8—N3—C7	119.64 (19)	C13—C12—H12A	119.4
N1—C1—C2	132.2 (2)	C14—C13—C12	119.6 (2)
N1—C1—C6	105.33 (18)	C14—C13—H13A	120.2
C2—C1—C6	122.4 (2)	C12—C13—H13A	120.2
C3—C2—C1	117.0 (2)	C13—C14—C15	121.3 (2)
C3—C2—H2A	121.5	C13—C14—H14A	119.4
C1—C2—H2A	121.5	C15—C14—H14A	119.4
C2—C3—C4	121.4 (2)	C14—C15—C16	121.6 (2)
C2—C3—H3A	119.3	C14—C15—C10	119.8 (2)
C4—C3—H3A	119.3	C16—C15—C10	118.6 (2)
C5—C4—C3	121.2 (2)	C17—C16—C15	122.0 (2)
C5—C4—H4A	119.4	C17—C16—H16A	119.0
C3—C4—H4A	119.4	C15—C16—H16A	119.0
C4—C5—C6	118.6 (2)	C16—C17—C18	120.3 (2)
C4—C5—H5A	120.7	C16—C17—H17A	119.9
C6—C5—H5A	120.7	C18—C17—H17A	119.9
N2—C6—C5	130.1 (2)	O1—C18—C9	122.3 (2)
N2—C6—C1	110.63 (19)	O1—C18—C17	116.6 (2)
C5—C6—C1	119.3 (2)	C9—C18—C17	121.1 (2)
N2—C7—N1	114.16 (19)	N1—C19—H19A	107 (3)
N2—C7—N3	128.24 (19)	N1—C19—H19B	112 (2)
N1—C7—N3	117.6 (2)	H19A—C19—H19B	109 (4)
N3—C8—C9	121.3 (2)	N1—C19—H19C	108 (2)
N3—C8—H8A	119.3	H19A—C19—H19C	104 (4)
C9—C8—H8A	119.3	H19B—C19—H19C	117 (4)
C18—C9—C8	119.9 (2)	N1—C19—H19Z	109 (2)

C18—C9—C10	118.7 (2)	N1—C19—H19Y	105 (3)
C8—C9—C10	121.4 (2)	H19Z—C19—H19Y	109 (4)
C11—C10—C15	117.1 (2)	N1—C19—H19X	108 (3)
C11—C10—C9	123.52 (19)	H19Z—C19—H19X	113 (4)
C15—C10—C9	119.3 (2)	H19Y—C19—H19X	112 (4)
